# Misdiagnosis of a multi-organ involvement hematogenous disseminated tuberculosis as metastasis: a case report and literature review

**DOI:** 10.1186/s40249-020-00681-8

**Published:** 2020-06-09

**Authors:** Tian-Xing Hang, Gang Fang, Yan Huang, Chun-Mei Hu, Wei Chen

**Affiliations:** 1 Department of Tuberculosis, the Second Hospital of Nanjing, Nanjing University of Chinese Medicine, 1-1 Zhongfu Road, Gulou District, Nanjing, Jiangsu Province, 210003 China; 2 Clinical Research Center, the Second Hospital of Nanjing, Nanjing University of Chinese Medicine, 1-1 Zhongfu Road, Gulou District, Nanjing, Jiangsu Province, 210003 China

**Keywords:** Misdiagnosis, Hematogenous disseminated tuberculosis, Metastasis, Pulmonary nodule

## Abstract

**Background:**

Tuberculosis (TB) is a great mimicker and diagnostic chameleon, and prone to be diagnosed as malignancy. Even though many reports have described the differences between pulmonary TB and lung cancer, the atypical systemic hematogenous disseminated TB (HDTB) is very rare and more confusing in clinical practice.

**Case presentation:**

A 73-year-old man, HIV-negative, was hospitalized to the local county hospital because of chest pain, low-grade fever, asthenia, anorexia and weight loss for the pasting two months. The CT findings of the two lungs showed multiple round or round-like nodules of different sizes, with clear boundaries and partial fusion. The level of serum CA19–9 was significantly higher than normal, and progressively increased. There were multiple enlarged lymph nodes in the neck, mediastinum, abdominal cavity and pelvic cavity. The symptoms were diagnosed as hematogenous spread of gastrointestinal tumor in the local county hospital. However, when transferred to our provincial hospital, through comprehensive dynamic analysis, this patient was diagnosed as atypical systemic HDTB, no cancer at all. Through routine anti-TB therapy for one year, the patient was recovered very well at the follow-up of half year after withdrawal.

**Conclusions:**

In the past, most TB misdiagnosis cases involved in single organ and were finally confirmed through invasive examination. This case enriched clinical experiences in the diagnosis of atypical HDTB. We encouraged clinicians to establish a dynamic thinking for diagnosis and treatment and emphasized the value of biopsy and ^18^F-FDG-PET in distinguishing TB and cancer.

## Background

Tuberculosis (TB) is a huge threat for public health in China, which is within the “high-burden country” list [1]. Even though enormous effort has been taken, treatment and control of TB still face many challenges. Detection and diagnosis of *Mycobacterium tuberculosis* (MTB) is difficult in most case. What’s worse, TB is a great mimicker and diagnostic chameleon, and prone to be diagnosed as malignancy, and vice-versa, because the radiographic characteristics and clinical symptoms of the two diseases are very similar [2–5]. Since cancer usually has worse prognosis and costs more medical resources compared with TB, misdiagnosis surely increases much more psychological stress and economical burden to the real TB patients. Furthermore, treatment strategy is totally different between neoplasm and TB. In such case, clinicians are placed into a dilemma to make a definite diagnosis, and are even at risk to become a “misdiagnosis murder” [6]. Thus, it’s pivotal to describe and summarize the nuances between neoplasm and TB through more case reports. Here, we described a very unusual case that a 73-year-old immunocompetent man with hematogenous disseminated TB (HDTB) was misdiagnosed as metastasis with multiple organs involvement.

## Case presentation

### Lung metastasis

**Fig. 1:**
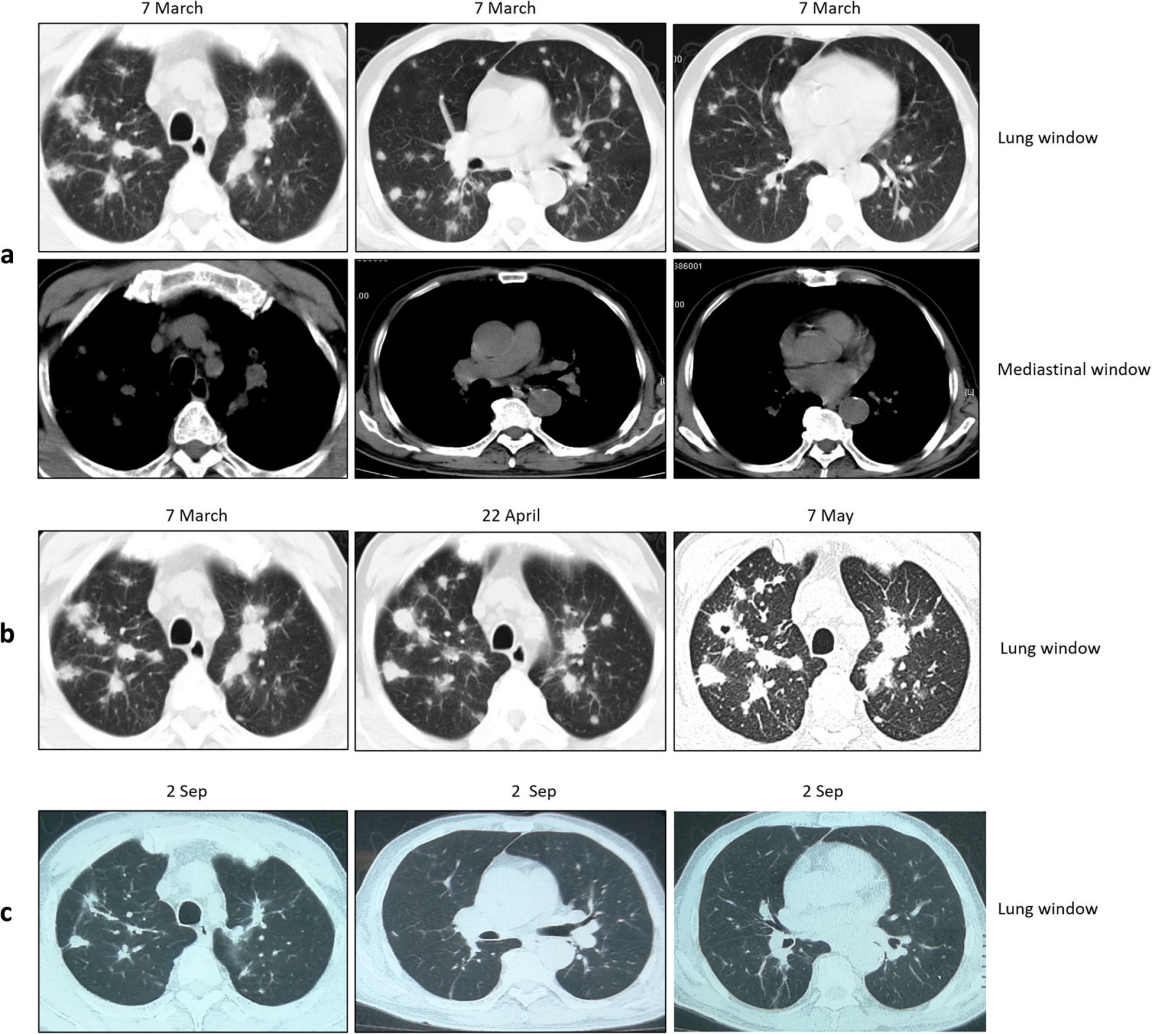
Thorax CT scan at different time points displayed evolution of lesions. **a** At admission, the thorax CT revealed multiple round or round-like nodules with variable size scattered throughout both lungs. **b** Development and evolution of lung lesions. The lung CT scan showed that pulmonary nodules gradually evolved with signs of fibrous tissue hyperplasia, such as cords, long burrs and fusion. **c** After four months of anti-TB treatment, thorax CT scan showed obvious absorbance of the lung lesions

**Fig. 2:**
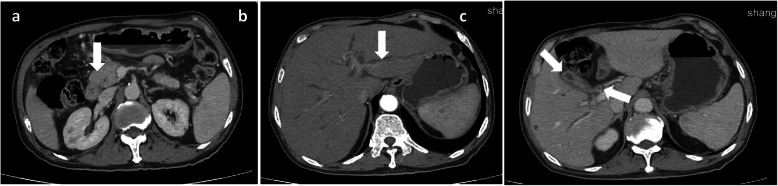
Abdominal enhancement CT suggested lesions in the intestinal tract. **a** There was a mass at the pancreatic head, indicated by arrow. **b** Intrahepatic bile duct was dilated, indicated by arrow. **c** The gallbladder wall (left arrow) and the wall of the upper end of the common bile duct (right arrow) were thicken

On 7 March, 2018, a 73-year-old man, HIV-negative, was hospitalized to the local county hospital for 2-month chest pain, low-grade fever, asthenia, anorexia and weight loss. The patient had history of type II diabetes mellitus with regular administration of gliclazide, but efficacy was poor. Physical examination revealed body temperature 36.7 °C, pulse rate 96 beats/min, blood pressure 133/83 mmHg, respiratory rate 20/min, oxygen saturation 96% and mildly yellow skin and sclera. Respiratory system, cardiovascular system, neurological system and abdomen were normal. Laboratory examination showed anemia (hemoglobin 114 g/L), abnormal liver function (alanine aminotransferase 57 U/L, aspartate aminotransferase 48 U/L, γ-glutamyl transpeptidase 818 U/L, alkaline phosphatase 671 U/L, total bilirubin 44 μmol/L) and elevated cancer antigen 19-9 (CA19-9, 165 U/ml). The interferon-γ release assay test was negative. The skull computerized tomography (CT) showed lacunar infarction in bilateral basal ganglia. The thorax CT revealed multiple round or round-like nodules with variable sizes scattered throughout both lungs, suggestive of metastatic lung disease (Fig. 1A). Abdominal enhancement CT showed that a mass at the pancreatic head was obviously strengthened in the arterial phase, and the pancreatic duct and intrahepatic bile duct was slightly dilated (Fig. 2). Taken together, all examination results were strongly suspected of lung metastasis, which was originated from the pancreatic head or intestinal origin. However, TB infection could not be excluded completely. The patient denied further biopsy, and chose to accept experimental anti-TB therapy with rifampin and isoniazid. After one month, chest CT showed that lung lesions were not absorbed. Laboratory test showed that CA19-9 sharply increased, up to 1167 U/ml. Sputum smear for acid-fast bacilli (AFB) was negative. The doctors in the local county hospital excluded TB infection and proposed gastrointestinal malignant tumors combined with lung metastasis. The patient family was in deep sorrow and prepared to give up.

### Hematogenous disseminated TB

**Fig. 3:**
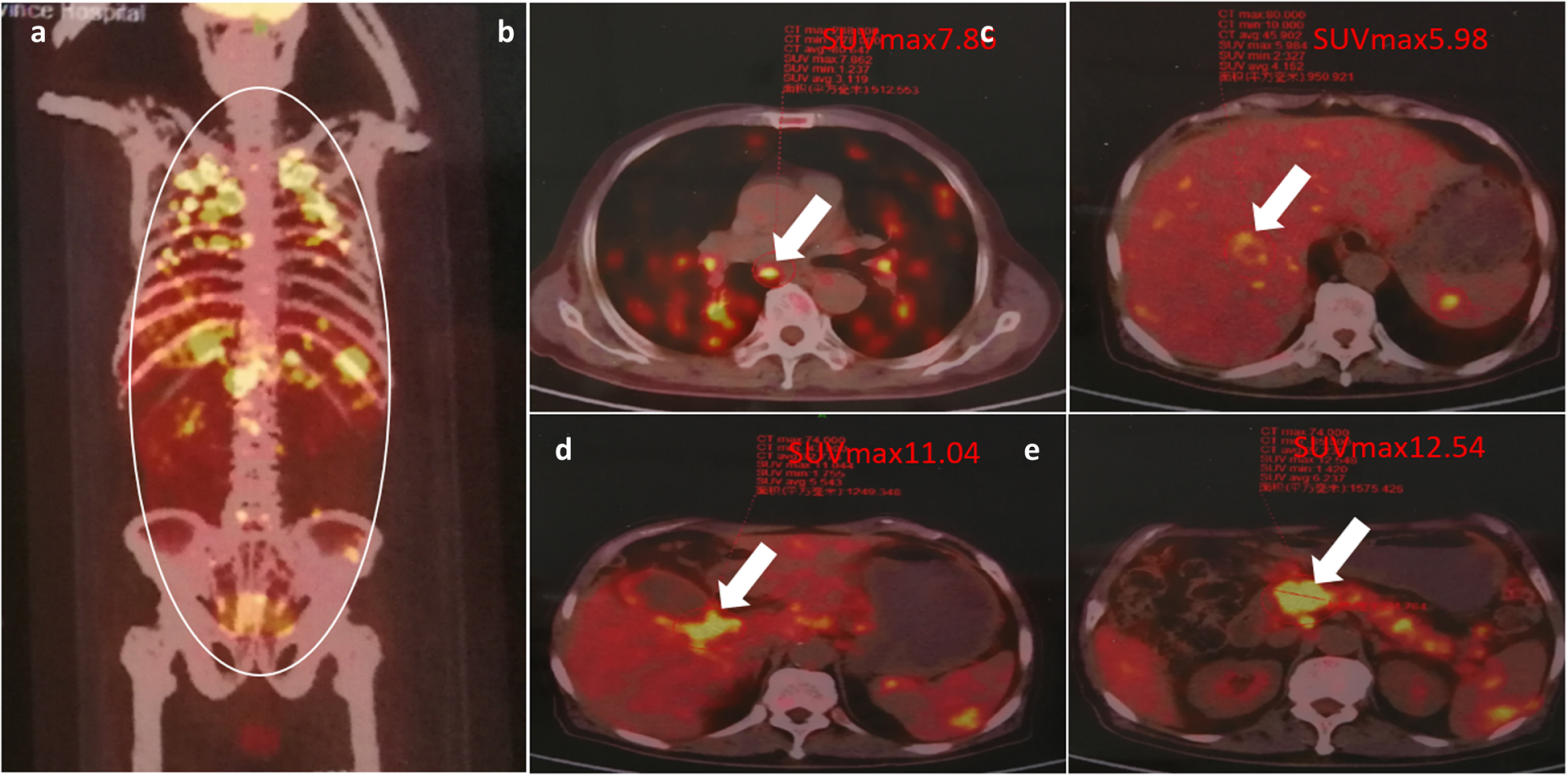
FDG-PET showed high uptake at different foci. **a** Foci in the whole body were defined by a circle. The yellow bright spots represented high uptake of ^18^F-FDG by local tissues. The maximum standard uptake value in different organs were shown, including lung (**b**, 7.86), liver (**c**, 5.98), gallbladder neck (**d**, 11.04), pancreatic head mass (**e**, 12.54)

**Fig. 4:**
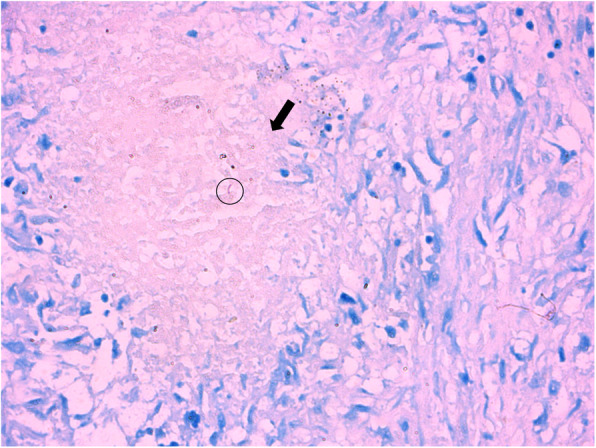
Puncture biopsy of left lung upper lobe revealed coagulative necrosis combined with granulomatous inflammation with hematoxylin-eosin staining (black arrow). Ziehl-Neelsen staining detected acid-fast bacilli (black circle) and Periodic Acid-Schiff staining was negative. Magnification × 200

On 7 May, 2018, his daughter persuaded the patient to the provincial hospital for further diagnosis. Laboratory examination revealed hyperbilirubinemia (total bilirubin 70 μmol/L), CA19-9 832 U/ml, cancer antigen 125 141 U/ml, neuron-specific enolase 22.98 ng/ml, angiotensin-converting enzyme 178 U/L and serum (1,3)-β-D-glucan 235 pg/ml. ^18^F-fluorodeoxyglucose positron-emission tomography (^18^F-FDG-PET) scan showed focal high uptake in the multiple organs, including lung, liver, pancreas, spleen, gallbladder neck (Fig. 3), which suggested benign disease (especially TB). The patient accepted the endoscopic retrograde cholangiopancreatography to relieve hyperbilirubinemia, which was caused by bile duct obstruction. Biopsy from brushed biliary cell indicated no malignant tumor. A CT scan-guided transthoracic needle biopsy of the nodule at the left upper lobe was performed and the histopathology showed coagulative necrosis combining with granulomatous inflammation. AFB staining was positive and Periodic Acid-Schiff staining was negative (Fig. 4). In the meanwhile, qPCR for MTB DNA showed 4900 copy/ml in the sputum. Thus, the diagnosis of TB infection was definite. The patient then accepted anti-TB regimen, including isoniazid (300 mg/once a day [QD]), rifampicin (450 mg/QD), ethambutol (750 mg/QD), pyrazinamide (1500 mg/QD), levofloxacin (600 mg/QD) for three months, followed by isoniazid (300 mg/QD) and rifampicin (450 mg/QD) for another nine months.

On 2 September, 2018, almost four months after hospitalization to our hospital, the thorax CT revealed that the lung lesions were absorbed significantly (Fig. 1B). After one year of treatment, continuous clinical and radiological improvement was observed, and the drugs were then withdrawn. The patient was asymptomatic at the follow-up of half year after withdrawal.

## Discussion

### Characteristics of TB misdiagnosis cases

We conducted literature search in NCBI PubMed database with keywords “tuberculosis” and “cancer” or “tumor” from 2016 to 2019. Totally, 23 hits were retrieved about TB misdiagnosis [7–28], including 1 summary analysis and 22 case reports. Half of 22 patients were young adults, with average age under 40. To our interest, none of these 22 patients had underlying diseases at admission except 2 with oncology history, 1 with familial Mediterranean fever, and 1 just subjected to kidney transplantation. It’s noted that 15 (68.2%) of the 22 cases involved in single organ, with lung the most involved organ (54.5%). Of the 22 cases, 19 (86.4%) were confirmed by invasive examination, like biopsy of the lesion. In summary, the clinical manifestations of some TB cases are non-specific, which are most likely misdiagnosed as tumor.

### Differences of pulmonary modules between lung metastasis and HDTB

Pulmonary nodules refer to the round intrapulmonary lesions with less than 3 cm in diameter [29]. Multiple nodules in the lung are one of the most common clinical imaging presentations, which appear in a variety of diseases, such as lymphangitis carcinomatosa, silicosis, and bronchioloalveolar carcinoma [30]. In particular, multiple small pulmonary nodules are frequently observed in both lung metastasis and HDTB [31, 32]. Besides, some chronic inflammation and unusual lymphoma involving in the lungs, could also display multiple pulmonary nodules [33]. The imaging manifestations of those diseases are so similar that a definite diagnosis is difficult to make [34]. The differences of nodule characteristics between lung metastasis and HDTB are summarized in Table 1. In our case, lung CT showed multiple round nodules of different size, with clear boundaries and partial fusion. Abdominal CT showed slight dilatation of intrahepatic bile duct and pancreas, enlargement of pancreatic head, multiple low-density foci in spleen.

**Table 1:** Comparison of nodule characteristics between lung metastasis and hematogenous disseminated tuberculosis

	Lung metastasis [35, 36]	Hematogenous disseminated tuberculosis (HDTB) [37, 38]	
**Sources**	Hematogenous	Hematogenous	
**Cancer history**	Yes	No	
**Anatomical distribution**	Scatter within two lung fields, but mostly distribute in the middle and lower lung field and surround subpleural lung tissues.	**Acute HDTB**	**Subacute or chronic HDTB**
		Randomly and evenly distribute in both lung fields	Randomly scatter in the two lung fields without uniformity the upper middle lung is more common
**Nature**	Solid density, ground glass density, or mixed density	Uniform density	Uneven density, may display tuberculous globules, or tuberculoma
**Size**	Variable	Uniform size, more miliary nodules	Variable
**Boundary**	Most of nodules have clear boundaries, but part of them not, showing cavity or ground glass opacity	Clear	Not clear, may display cavity or ground glass opacity
**Accompanying symptoms**	Mediastinal lymph node enlargement and pleural effusion	Mediastinal lymph node enlargement	

### Nonspecificity of CA19-9

CA19-9 level was significantly higher than normal, and progressively increased. It is known that, CA19-9 is synthesized by pancreatic and biliary ductal cells, as well as gastric, colon, endometrial and salivary epithelia. Only small amount of CA19-9 is present in serum [39]. CA19-9 is considered as the marker for pancreatic cancer in clinical practice. However, this indicator has the disadvantage of low specificity [39, 40]. Overexpression of CA19-9 was observed in several benign gastrointestinal disorders, which may be relevant to glycan mediated cell-cell interactions in mucosal immunity [40]. Additionally, higher level of CA19-9 was found in the pancreatic tuberculosis [41, 42]. In our case, sharply increased CA19-9 also played a role of misleading.

### Clues to make definite diagnosis

When we looked into these symptoms with comprehensive dynamic analysis, we found that this patient had anemia and his white blood cells was not high, indicating that he was in the course of chronic infection (our clinical experience). There were multiple pulmonary nodules, most of which distributed along with the bronchovascular bundle. Some nodules were not very regular. Pulmonary nodules in the upper lung showed a trend of enlargement, fusion and infiltration. In addition, signs of fibrous hyperplasia such as strips and long burrs appeared in the process of evolution (Fig. 1C). We gradually thought it was more likely to be inflammatory granuloma lesions, instead of malignant tumor. At the other hand, abdominal CT suggested thickening of the gallbladder wall and uniform thickening of the upper-end wall of the common bile duct. Although the head of the pancreas was enlarged, it still had regular shape, clear boundary, and consistent enhancement with the whole pancreas body. All these signs also suggested chronic inflammation. Additionally, in this case, the maximum standard uptake value (SUVmax) of PET CT imaging for pulmonary nodules was 7.86, located between 2.65 and 10.9, which probably indicated benign lesions. The value of PET CT in evaluating the efficacy of TB treatment, especially extrapulmonary TB, has been widely recognized [23]. Unfortunately, given its high cost, it cannot be widely used in China at present. The patient also refused to accept re-examination by PET CT for this reason. Otherwise, the effect of individualized chemotherapy regimen can be better evaluated through the changes in pathological metabolism. Finally, puncture biopsy of lung confirmed pulmonary TB. Through anti-TB treatment, the patient was cure, which in turn supported our diagnosis.

## Conclusions

The patient turned out to be atypical systemic HDTB, not cancer at all. Such atypical HDTB was relatively rare in clinical practice. We encouraged clinicians to keep dynamic thinking of the disease in mind and know more special signs of rare diseases.

## Data Availability

Data relating to this study are contained and presented in this document. Other materials are available from the corresponding authors on reasonable request.

## Abbreviations

TB: Tuberculosis
MTB: *Mycobacterium tuberculosis*
HDTB: Hematogenous disseminated TB
CT: Computerized tomography
AFB: Acid-fast bacilli
^18^F-FDG-PET: ^18^F-fluorodeoxyglucose positron-emission tomography
QD: Once a day
